# Daily Physical Activity Patterns and Their Association With Health-Related Physical Fitness Among Aging Workers

**DOI:** 10.1093/geroni/igaa057.2866

**Published:** 2020-12-16

**Authors:** Sari Stenholm, Anna Pulakka, Tuija Leskinen, Jaana Pentti, Olli Heinonen, Annemarie Koster, Jussi Vahtera

**Affiliations:** 1 University of Turku, Turku, Finland; 2 National Institute for Health and welfare, Helsinki, Uusimaa, Finland; 3 University of Turku, Turku, Varsinais-Suomi, Finland; 4 University of Helsinki, Helsinki, Uusimaa, Finland; 5 Maastricht University, Maastricht, Limburg, Netherlands

## Abstract

This study aimed to identify accelerometer measured daily physical activity patterns and to examine how they associate with health-related physical fitness among 258 participants (mean age 62.4 years, SD 1.0) from the Finnish Retirement and Aging Study. Wrist-worn ActiGraph accelerometer was used and health-related physical fitness measures included body composition, cardiorespiratory fitness and muscular fitness. Based on latent class trajectory analysis, six different patterns of daily physical activity was identified on workdays and two on days off. Having low activity throughout the workday was associated with poorest health-related physical fitness, whereas a combination of low or moderate activity during working hours and increase of activity level in the evening was associated with most favorable body composition and better physical fitness compared to the other trajectories. In conclusion, a large variation in the workday physical activity patterns and health-related physical fitness was observed among aging workers.

